# A proposal for a multivariate quantitative approach to infer karyological relationships among taxa

**DOI:** 10.3897/CompCytogen.v8i4.8564

**Published:** 2014-12-10

**Authors:** Lorenzo Peruzzi, Fahim Altınordu

**Affiliations:** 1 Department of Biology, Unit of Botany, Pisa University, Via L. Ghini 13, 56126 Pisa, Italy; 2 Department of Biology, Science Faculty, Selçuk University, Konya, Turkey

**Keywords:** Comparative cytogenetics, cytotaxonomy, karyotype asymmetry, karyotype variation, PCoA

## Abstract

Until now, basic karyological parameters have been used in different ways by researchers to infer karyological relationships among organisms. In the present study, we propose a standardized approach to this aim, integrating six different, not redundant, parameters in a multivariate PCoA analysis. These parameters are chromosome number, basic chromosome number, total haploid chromosome length, M_CA_ (Mean Centromeric Asymmetry), CV_CL_ (Coefficient of Variation of Chromosome Length) and CV_CI_ (Coefficient of Variation of Centromeric Index). The method is exemplified with the application to several plant taxa, and its significance and limits are discussed in the light of current phylogenetic knowledge of these groups.

## Introduction

Chromosomes, especially those of plants, have been efficient material for almost every kind of cytogenetic research (Guerra 2005, 2012). The genetic information of an organism is transferred via chromosomes, and changes in their number (e.g. polyploidy, dysploidy) and structure (rearrangements such as inversions, deletions, or translocations) are important contributors to plant evolution and speciation (Levin 2002, Doyle et al. 2004, Schubert 2007, Leitch and Leitch 2008, Weiss-Schneeweiss et al. 2009). Since the putative discovery of a constant species-speciﬁc chromosome number by Strasburger (1910), several times researchers posed the question, whether basic karyotype structure might provide information about the systematic position of a species (Venora et al. 2008). As a result, vast amounts of data on chromosome number have been collected until now (Stace 2000, Garbari et al. 2012) and chromosome data are constantly used for karyosystematic purposes. More recently, efforts to process this huge quantity of chromosome numbers accumulated in literature have been made, producing interesting results (Peruzzi et al. 2011, 2012, 2014, Bedini et al. 2012, Góralski et al. 2013, 2014). However, it is well known that chromosome numbers alone are not sufficient to exactly trace the evolutionary history of a group (Weiss-Schneeweiss and Schneeweiss 2003). Also, when considering some genera with many species, the ecological and the morphological data may not be an efficient tool to provide a clear representation of the systematic relationships between species. In these cases cytotaxonomy (or comparative cytogenetics), together with molecular data, can be an effective tool and it can allow a more accurate knowledge of the relationships (Coutinho 1952, Dewey 1984, Venora et al. 2008). In such cases, more detailed information about the karyotype is essential besides the chromosome number.

The karyotype of a species is generally subject to little variation and it is generally assumed that two similar species can be different for a number of chromosome rearrangements correlated with phylogenetic distance among them (Stebbins 1966, Venora et al. 2008). Karyomorphological traits are evaluated by many authors as important taxonomic characters which not only provide additional characters but also allow conclusions about evolutionary events in the group of interest (Greilhuber and Speta 1978, Greilhuber 1982, Cerbah et al. 1998, Weiss-Schneeweiss and Schneeweiss 2003). A karyotype clarifies the phenotypic aspects of the chromosome complement of a species in terms of number, size, arm ratio, centromere position, and other basic landmark features of its chromosomes (Levin 2002). In recent years, in the light of the great positive impact of the molecular phylogeny, the knowledge on the chromosome complement is still a fundamental aid to evaluate the phylogenetic relationships among taxa (Garbari et al. 2012 and literature cited therein). The karyotype asymmetry is a good expression of the general morphology of plant chromosomes. It is therefore very important to have a uniform system to compare karyotypes on correct statistical grounds (Paszko 2006). The position of centromere and the relative chromosome size are the two most important karyotype features which allowed reasonable assessment of chromosomal affinities based on the concept of symmetry (Lavania and Srivastava 1999). Hence the use of statistically correct parameters as characters for the reconstruction of karyological relationships is fundamental. Some authors also tried to reconstruct phylogenetic relationships using only the highest possible number of karyological parameters (Caputo et al. 2013 and literature cited therein). However, until now two main problems were, more or less consciously, encountered by researchers: a) a lack of agreement in which karyotype asymmetry parameters have to be used, often leading to their misuse (e.g. redundancy etc.); b) the use of taxon-specific parameters, not of general applicability (for instance the comparison of each chromosome pair in a karyotype, which can be carried out only among closely related taxa with equal chromosome number). Concerning karyotype asymmetry, we think that the revisions of Paszko (2006), Zuo and Yuan (2011) and Peruzzi and Eroğlu (2013) were decisive, in definitely showing how and what to measure (see beyond, in Materials and methods, for more details). Despite this, many researchers – even in the very last year – continued to use outdated and often not statistically correct parameters to quantify karyotype asymmetry (Gao et al. 2012, Eroğlu et al. 2013, Wang et al. 2013, Altınordu et al. 2014, Morales et al. 2014, De Oliveira et al. 2014, Jafari et al. 2014, Chen et al. 2014). In addition, a number of basic karyological parameters (besides karyotype asymmetry) are of general applicability and can be compared among taxa: chromosome number, basic chromosome number (*x*, as defined by Peruzzi 2013), and total length of chromosomes (which is a rough proxy of genome size; Peruzzi et al. 2009).

Hence, the aims of our study were (1) to propose a standardized use of basic karyological characters as a valid, of general use, complement to other source of systematic data to understand the relationships among taxonomic groups as families, tribes, genera, sections and species, and (2) to demonstrate the using of this new quantitative method in cytotaxonomy in selected groups, for which data were available in literature.

## Materials and methods

### Data source

The data about Smilacaceae, Liliaceae and its tribes and genera were derived by Kong et al. (2007) and by the supplementary material published along with Peruzzi et al. (2009), Gao et al. (2012), and by Peruzzi (2012), concerning specifically the genus *Gagea* Salisbury, 1806. For *Cyananthus* Wallich ex Bentham, 1836 (Campanulaceae) and for *Crocus* Linnaeus, 1753 ser. *Verni* Mathew, 1982 (Iridaceae), the data were derived by the recent papers by Chen et al. (2014) and Harpke et al. (2014), respectively. Most of these papers report also information on the phylogenetic relationships among groups (for *Cyananthus* available in Zhou et al. 2013), as inferred from molecular systematic studies. All the datasets are available as supplementary material.

### Karyological parameters

To determine the karyological relationships among taxa, we used chromosome number (2*n*), basic chromosome number (*x*), and other basic karyomorphological characters such as genome size, grossly estimated as total haploid length of the chromosome set, THL (Peruzzi et al. 2009). Also karyotype symmetry indices were used, such as M_CA_ (Mean Centromeric Asymmetry) which gives a measure of intrachromosomal asymmetry, and CV_CL_ (Coefficient of Variation of Chromosome Length) which gives a measure of interchromosomal asymmetry, together with CV_CI_ (Coefficient of Variation of Centromeric Index), which gives a measure of centromere position heterogeneity (Paszko 2006; Zuo and Yuan 2011, Peruzzi and Eroğlu 2013). For a karyotype, M_CA_ is calculated as the mean (L-S)/(L+S) ×100 where, for each chromosome, L is the length of long arm and S is the length of short arm; CV_CL_ as the standard deviation of (L+S) divided by the mean (L+S) ×100; CV_CI_ as the standard deviation of S/(L+S) divided by the mean S/(L+S) ×100. These three parameters estimate quantitatively three different features of a karyotype, so that any redundancy of data is avoided. Moreover, they were shown to be the only quantitative parameters correct on statistical grounds (Peruzzi and Eroğlu 2013). For these reasons, other parameters proposed earlier to estimate the intrachromosomal (TF%, AsK%, AsI%, Syi, A_1_, CG; for details and references see Peruzzi and Eroğlu 2013) or the interchromosomal asymmetry (Rec, R; for details and references see Peruzzi and Eroğlu 2013) were discarded. The same applied also to semi-quantitative methods such as that of Stebbins (1971) or to indices trying to summarize both kind of asymmetries (intra- and inter-chromosomal) in a single value (i.e. DI, AI; for details and references see Paszko 2006, and Peruzzi et al. 2009 for criticisms). Also karyomorphometric measurements of single chromosome pairs (as for instance those used by Caputo et al. 2013 and in previous works of the same research team) were not considered, to guarantee a general applicability of the method independent from chromosome number.

Other karyological characters might have been used, such as number of 45S and 5S sites or “best practice” genome size estimations, but this kind of data is not yet widespread (Roa and Guerra 2012; Garcia et al. 2012, 2014a, 2014b) and would also limit the applicability of the method.

### Data analysis

Since our main objective was to highlight correctly karyological relationships among objects (e.g. single accessions) and not to form groups, we avoided multivariate classification techniques such as cluster analysis etc. and focused on a general ordination method as PCoA (Principal Coordinate Analysis). In cases where specific *a priori* grouping hypotheses (based on independent sources of systematic data) needed to be tested, this approach was complemented by subjecting the same data matrix to DA (Discriminant Analysis). To perform PCoA, a similarity matrix was created using Gower’s (1971) general coefficient similarity to summarize relationship among accessions (Sneath and Sokal 1973), which can be used directly with a mixture of character types (binary, qualitative, and quantitative characters) as well as taking into account missing values (St-Laurent et al. 2000). To perform these kind of analyses, the software Past 3.03 (Hammer et al. 2001, Hammer 2013), freely available online, was used.

## Results

### Testing the new approach at family level

**Figure 1.**
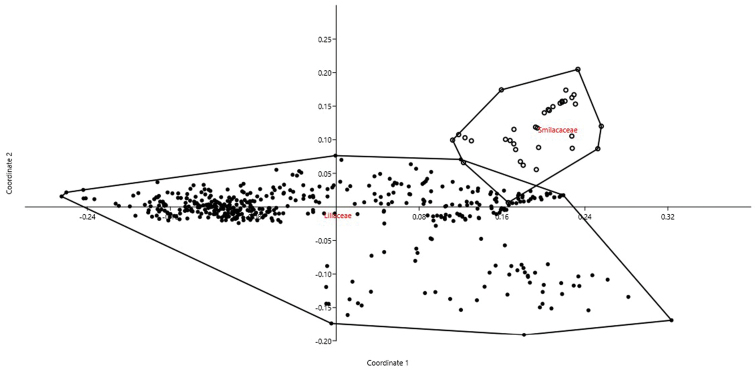
PCoA for Liliaceae and Smilacaceae based on 6 quantitative karyological parameters (Axis 1 vs. Axis 2).

We analyzed 434 accessions for Liliaceae and 35 accessions for Smilacaceae by PCoA (cumulative variance explained by the first two axes: 54.21%). Only a modest overlap among the two families was evident (Fig. 1). Indeed, DA correctly attributed objects (accessions) to the two families in 95.24% of cases (jackknifed). The most important characters in recognizing the two families as distinct resulted THL, CV_CI_, and M_CA_.

### Testing the new approach at tribe level

**Figure 2.**
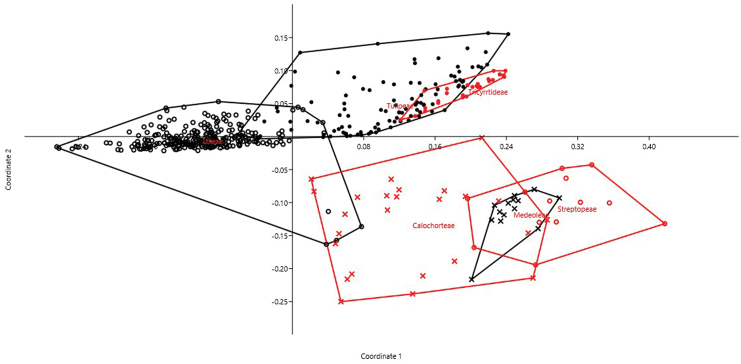
PCoA for Liliaceae tribes based on on 6 quantitative karyological parameters (Axis 1 vs. Axis 2).

Within Liliaceae, 103 accessions for Tulipeae tribe, 252 accessions for Lilieae tribe, 14 accessions for Medeoelae tribe, 13 accessions for Streptopeae tribe, 27 accessions for Tricyrtideae tribe and 25 accessions for Calochorteae tribe were analyzed by PCoA (cumulative variance explained by the first two axes: 53.96%). Also in this case, the accessions belonging to the same tribe clearly tend to cluster together (Fig. 2). Indeed, DA correctly attributed objects (accessions) to the two families in 93.97% of cases (jackknifed). The most important characters in recognizing the two families as distinct resulted THL, CV_CL_, and M_CA_.

### Testing the new approach at genus level

**Figure 3.**
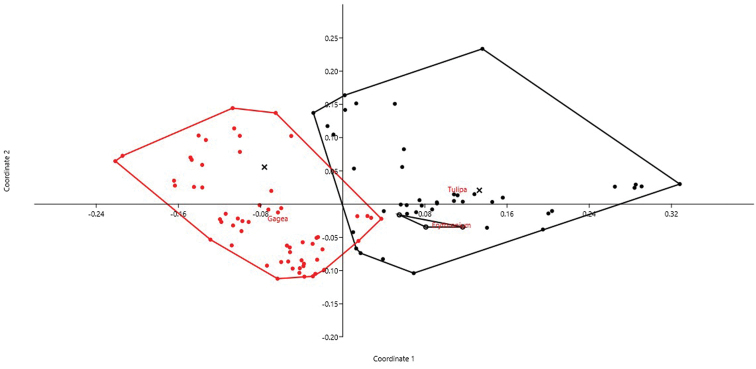
PCoA for Tulipeae genera based on 6 quantitative karyological parameters (Axis 1 vs. Axis 2). The two *Amana* accessions are represented by the “×” symbol.

Within Liliaceae tribe Tulipeae, *Erythronium* Linnaeus, 1753 (3), *Tulipa* Linnaeus, 1753 (42), *Amana* Honda, 1935 (2), *Gagea* (56) accessions were analyzed by PCoA (cumulative variance explained by the first two axes: 48.3%). The isolated position of *Gagea* respect with other genera was particularly evident (Fig. 3). The DA, restricted to *Gagea* and *Tulipa*, correctly attributed objects (accessions) to the two genera in 94.12% of cases (jackknifed). The most important characters in recognizing the two families as distinct resulted THL, M_CA_, and CV_CL_.

### Testing the new approach at section level

**Figure 4.**
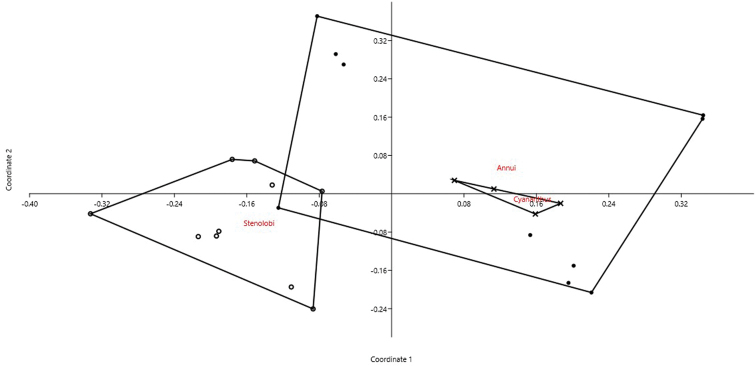
PCoA for *Cyananthus* accessions based on 6 quantitative karyological parameters (Axis 1 vs. Axis 2).

**Figure 5.**
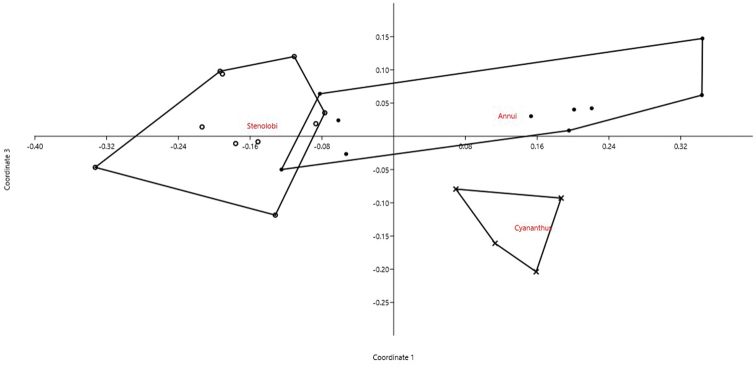
PCoA for *Cyananthus* accessions based on 6 quantitative karyological parameters (Axis 1 vs. Axis 3).

We analyzed 24 accessions belonging to three sections (*Annui*, *Cyananthus*, and *Stenolobi*) representing 15 species of the genus *Cyananthus* (Campanulaceae) by PCoA (cumulative variance explained by the first two axes: 65.52%). We can see a certain overlap among all sections, with *Stenolobi* seemingly more isolated and *Cyananthus* forming a homogeneous group within of *Annui* (Fig. 4). However, when the first axis is plotted against the third one, also these two sections appear well separated (Fig. 5). Indeed, DA correctly attributed objects (accessions) to the three sections in 87.5% of cases (jackknifed). In this case, the most important characters in recognizing the three sections resulted 2*n*, CV_CI_, and THL.

### Testing the new approach for relationships among closely related species

**Figure 6.**
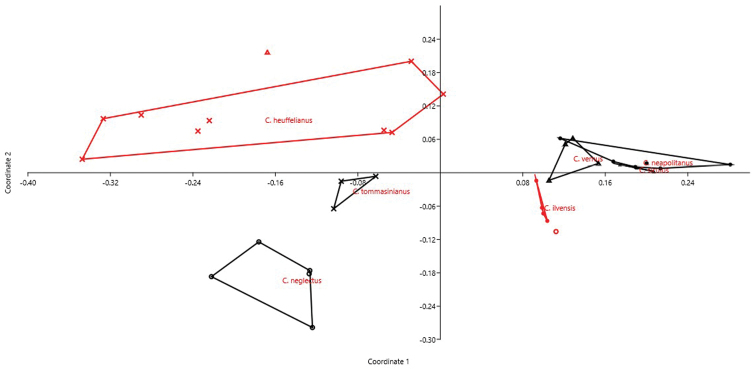
PCoA for *Crocus* accessions based on 6 quantitative karyological parameters (Axis 1 vs. Axis 2). The red circle and the red triangle depict the single accessions of *Crocus etruscus* and *Crocus kosaninii*, respectively.

We analyzed 36 accessions belonging to nine species of *Crocus* ser. *Verni* (Iridaceae): *Crocus etruscus* Parlatore, 1858 (1), *Crocus heuffelianus* Herbert, 1847 (9), *Crocus ilvensis* Peruzzi et Carta, 2011 (4), *Crocus kosaninii* Pulević, 1976 (1), *Crocus neapolitanus* (Ker Gawler) Loiseleur-Deslongchamps, 1817 (6), *Crocus neglectus* Peruzzi et Carta, 2014 (5), *Crocus siculus* Tineo, 1832 (3), *Crocus tommasinianus* Herbert, 1847) (3) and *Crocus vernus* (Linnaeus) Hill, 1765 (4) (cumulative variance explained by the first two axes: 58%). We can see the accessions belonging to same species close each other (Fig. 6). DA correctly attributed objects (accessions) to each species in 69.44% of cases (jackknifed). The most important characters in recognizing the three sections resulted THL, CV_CL_, and M_CA_.

## Discussion

Our method allows to describe basic karyological relationships among taxa in a correct way, avoiding redundant data or the use of statistically not well founded parameters. Concerning the examples presented, there is always a certain degree of agreement among the information resulting from karyological multivariate analysis and the available phylogenetic information (used to form the groups highlighted in the PCoA and tested by means of DA). Liliaceae and Smilacaceae are sister families (Peruzzi et al. 2009 and literature cited therein), and despite their closeness show very modest overlap on karyological grounds. This is true also at tribe level within Liliaceae, albeit for instance Tricyrtideae are karyologically closer to Tulipeae, while on phylogenetic grounds they result an independent lineage (Peruzzi et al. 2009). This can be easily explained by the striking overall similarity in karyotype structure among *Gagea* (within Tulipeae) and Tricyrtideae, albeit chromosome numbers are different (*x* = 12 the former, *x* = 13 the latter; Peruzzi et al. 2009). As far infrageneric taxa are concerned, *Cyananthus* sections show a certain degree of karyological separation. Zhou et al. (2013) showed that sect. *Cyananthus* is sister to *Annui* + *Stenolobi*. Our data point towards a higher karyological affinity between *Annui* and *Stenolobi* (Figs 4 and 5), as already evidenced by Chen et al. (2014). PCoA, however, highlights a certain karyological heterogeneity within sect. *Annui*, which is partly close to *Cyananthus* and in part overlapping to *Stenolobi*. The accessions falling close to *Cyananthus* in the PCoA share the same basic chromosome number with the latter. Also the karyological relationships among the species of *Crocus* ser. *Verni*, as evidenced here, are fully congruent with the current systematic knowledge of the group (Harpke et al. 2014). In particular, *Crocus neapolitanus*, *Crocus siculus* and *Crocus vernus* resulted karyologically very closely related species and this is supported by available phylogeny. The resolution of karyological relationships is much better than that obtained by simply plotting karyotype asymmetry parameters against each other, as done by Harpke et al. (2014).

## Conclusions

For various reasons, researchers used until very recently outdated, wrong or redundant parameters in order to establish relationships among taxa. We propose here a standardized method, taking into account six quantitative parameters: 2*n* (somatic chromosome number), *x* (basic chromosome number), THL (total length of haploid chromosome set), CV_CI_ (Coefficient of Variation of Centromeric Index, measuring the heterogeneity in the centromere position), M_CA_ and CV_CL_ (Mean Centromeric Asymmetry and Coefficient of Variation of Chromosome Length, both measuring the karyotype asymmetry). We used a multivariate ordination approach (PCoA), eventually complemented by DA, if specific grouping hypotheses need to be tested. We think this method is best suited to establish karyological relationships, relationships, compared with classification approaches (i.e. clustering, used for instance by Caputo et al. 2013, Chen et al. 2014 and many others), which may be misinterpreted concerning their real significance (i.e. a dendrogram can resemble a phylogenetic tree). We applied our method to several taxa at various ranks from family to species, showing that the discriminatory power of karyological parameters is very variable among groups. As already highlighted by Siljak-Yakovlev and Peruzzi (2012) and Peruzzi and Eroğlu (2013), basic karyological data alone are not sufficient to definitely establish systematic and phylogenetic relationships among taxa, and should always be complemented by independent sources of systematic data. However, karyological data significantly contribute to understanding evolutionary relationships, jointly with morphological and molecular approaches. To this aim, our method is better than others because it is easy to use, based on correct, not redundant parameters of general use, and also because the data are treated with ordination and not classification techniques.

## Supplementary materials

Supplementary material 1 Karyological parameters available for the studied taxa Data type: measurement Explanation note: Excel file with three different worksheets (Liliaceae+Smilacaceae; *Cyananthus*; *Crocus* ser. *Verni*). This dataset is made available under the Open Database License (http://opendatacommons.org/licenses/odbl/1.0/). The Open Database License (ODbL) is a license agreement intended to allow users to freely share, modify, and use this Dataset while maintaining this same freedom for others, provided that the original source and author(s) are credited.Lorenzo Peruzzi, Fahim Altınordu
